# Quantitative study of cytotoxic T-lymphocyte immunotherapy for nasopharyngeal carcinoma

**DOI:** 10.1186/1742-4682-9-6

**Published:** 2012-03-07

**Authors:** Wang Shengjun, Guo Yunbo, Song Liyan, Li Jinming, Deng Qinkai

**Affiliations:** 1 Medical Apparatus and Equipment Department, Nanfang Hospital, Southern Medical University, Guangzhou, P. R. China; 2 Bioinformatics Laboratory, School of Basic Medical Sciences, Southern Medical University, Guangzhou, P. R. China

## Abstract

**Background:**

In clinical practice, the common strategy for immunotherapy of nasopharyngeal carcinoma (NPC) is to infuse cytotoxic T-lymphocyte (CTL) lines several times by intravenous injection, but it is difficult by laboratory research to investigate the relationship between treatment time-point, the amount of CTL added and the therapeutic effect. The objective of this study is to establish a mathematical model to study the therapeutic effect of different treatment time-points and amounts of CTL, and to predict the change in therapeutic effect when the percentage of EBV LMP2-specific CTL is increased from 10% to 20%.

**Results:**

The concentration of epidermal growth factor receptor (EGFR) in the tumor cell cytomembranes increases after CTL is added. Concurrently, there is a marked downward trend of the phosphorylated transforming growth factor-α (TGFα)-EGFR complex in the tumor cell cytomembranes, which indicates restriction of tumor growth after CTL immunotherapy. The relationships among the time of addition of CTL, the amount of CTL added, different CTL specificities for LMP2 and the increment rate k of the total number of tumor cells were evaluated.

**Conclusions:**

The simulation results quantify the relationships among treatment time-points, amount of CTL added, and the corresponding therapeutic effect of immunotherapy for NPC.

## Background

Epstein-Barr virus is present in virtually all poorly-differentiated and undifferentiated nasopharyngeal carcinomas (NPC) and the viral antigens expressed by the tumor provide potential targets for immunotherapy [1]. NPCs only express a restricted set of less immunogenic viral antigens (latency type II), namely EBNA-1, LMP-1 and LMP-2. The most likely target antigen for a CD8+ cytotoxic T-lymphocyte (CTL)-based therapy is LMP-2.

A pilot study using adoptive T cell therapy to treat NPC was reported in 2001 [2]. Autologous EBV-transformed B-lymphoblastoid cell line (LCL)-reactivated T cells were generated in vitro and used to treat four advanced cases of NPC. The use of autologous EBV-specific CTL for NPC has since been evaluated in two clinical trials [3,4]. Both studies demonstrated that autologous EBV-specific CTL is safe, induces LMP-2-specific immune responses, and is associated with an objective response and control of disease in advanced NPC. However, the EBV-specific CTL lines in these studies were generated by stimulation with EBV-LCL, which favored the outgrowth of CTL responses to the immunodominant EBNA-3 proteins rather than the subdominant EBV proteins LMP-1 and LMP-2 expressed in NPC. The anti-tumor response could be further enhanced by strategies that increase the specificities of CTL lines for the EBV latency II antigens expressed in NPC [1].

How can the success rate of immunotherapy for NPC be increased? Straathof et al. [4] pointed out that the CTLs being transferred may undergo only limited expansion in vivo, so strategies aimed at increasing the number of LMP1- and LMP2-specific T cells in the infusion product may be of value. Fewer than 10% of cells in the CTL lines mentioned above are LMP2-specific.

The common strategy is to infuse CTL lines several times by intravenous injection in clinical practice, but it is difficult to investigate the relationships among treatment time-point, amount of CTL added and therapeutic effect by laboratory research. The objective of this work is to establish a mathematical model to study the therapeutic effect of different treatment time-points and amounts of CTL, and to predict the change in therapeutic effect when the percentage of EBV LMP2-specific CTL is increased from 10% to 20%.

## Methods

The simulation toolkit used in this study is REPAST (Recursive Porous Agent Simulation Toolkit), which originated from the University of Chicago. Its original purpose was to build a manageable and flexible agent-based modeling (ABM) platform for social simulation [5]. The current Repast platform has been developed to Repast Simphony from Repast 3, in which the applications have been extended to natural science, especially biomedicine [6-8].

The ABM used in this study consists of two models. One is the ABM of tumor growth built by Athale et al. [9]. The other is Basic Immune Simulator (BIS), which is an agent-based model created to study the interactions between the cells of the innate and adaptive immune systems [10], and therefore to simulate the human immune system. These two models are both built on the platform of Repast 3. In order to collect data and create diagrams expediently, we input the two models into Repast Simphony. Some necessary modifications were made to accommodate the objective of the study.

The computer used in the simulation has an AMD athlon 64 bit dual-core processor with main frequency of 2.20 GHz and 2 GB RAM memory. It takes about 840 seconds to complete one simulation.

## Model of tumor growth

Our simulation made some appropriate modifications to the ABM of tumor growth built by Athale et al. [9]. A problem in the algorithm for nutrient diffusion was resolved. The experimental study by Thornburg et al. [11] indicated that the expressed EBV protein up-regulates EGFR expression in NPC by 12 fold, and this finding was incorporated in our simulation model.

The ABM is a multi-scale model that can be studied at different levels - molecular, microscopic (cell) and macroscopic (tissue). A cell in the model is divided into four layers: extracellular, membrane, cytoplasm and nucleus. The interactions of different molecules are shown in Figure 2 in reference [9]. The mass-balance differential equation takes the following generic form:

$$\frac{dX_{i}}{dt}=\alpha \cdot \;X_{i}-\;\beta \;\cdot \;X_{i}$$

where *X_i _*is the mass of the *i*^th ^molecule, *α *is the rate of synthesis or increase and *β *is the rate of degradation or removal of a molecular species. There is an equation for each molecule. Detailed equations and their parameters can be found in reference [9].

The change of each molecule in every agent (i.e. cell) is calculated at each iteration step of the simulation. Cells are assigned to be in one of three states, namely migration, proliferation or quiescence, according to the calculation. Migration means moving to neighboring grids. Whether or not a cell is in the migration state will be checked first, and if it is not, the algorithm will evaluate whether it is in the proliferation or quiescence state. The procedure is as follows:

① calculating $Mn\left[\left(PLC\gamma \right)\right]=\left[\frac{d\left[PLC\gamma \right]}{dt}\right]$, *Mn *is the change in amplitude of *PLC*γ in the n^th ^cell before and after an interation. *PLC*γ is active (phosphorylated, Ca-bound) phospholipase C-γ.

② if *Mn *>*σ_PLC_*, the cell's next state will be assigned as migration. The algorithm will judge further to which neighboring grids it moves.

③ calculating $P_{prolif}\left[\left(LR\right)\right]=\left(LR\right)-\sigma_{EGFR}$

where (*LR*) is the concentration of ligand-bound phosphorylated TGFα-EGFR complex in a cell, *σ_EGFR _*is the threshold, and *P_prolif _*indicates capacity for proliferation. This function is derived from experimental observations regarding cell proliferation in relation to an EGF-receptor threshold [12] and a model that relates receptor occupancy to percentage maximal

proliferation [13]. Specifically, these publications reported experimentally measured values of cell proliferation in response to EGFR occupancy for some human and rodent cell lines, and it was demonstrated that *σ_EGFR _*could reach 25% of the total receptor concentration [9].

If *Mn < σ_PLC _*and *P_prolif _*> 0, the cell will proliferate.

If *Mn < σ_PLC _*and *P_prolif _*< 0, the cell will be quiescent.

Conditions: the virtual environment consists of S = R^2 ^grids. R is the length of the lattice side and here R = 200. Each grid can be empty or be occupied by one cell. There are 100 tumor cells at the center of the grids when the model is initialized. There is a blood vessel, which is the initial location of the nutrient source from which glucose diffuses, vertical to the lattice at the center of the SE quadrant. The dynamic changes of concentrations of four molecules in/out of the tumor cells are recorded in the simulation. They are: EGFR in cytoplasm (marked as EGFR_i in figure), EGFR in membrane (EGFR_s), phosphorylated TGFα-EGFR complex in membrane (phos_TGFaEGFR_s), and extracellular TGF*α *(TGFα_ex). For each molecule, the calculation is carried out for cells inside the tumor tissue and on the tumor tissue surface. It is mainly in the quiescence state for the former and the proliferation/migration state for the latter.

### BIS model

BIS is an agent-based model for studying the interactions between cells of the innate and adaptive immune systems [10]. The BIS was created with three "zones" of activity to represent the separate locations in the body where interactions between cells take place during the course of an immune response. Zone 1 is the site where a tissue initially challenges the pathogen. Zone 2 is an abstract representation of a lymph node or the spleen, where lymphocytes reside and proliferate. Zone 3 is an abstract representation of the lymphatic and blood circulations - the conduits for the cells to travel in the immune system. Zone 3 was created to contain the agents that represent cells that must travel for indefinite (unknown) periods of time before arriving at their final destination, the site of pathological challenge (Zone 1). Thus, Zone 3 can be considered as the "rest of the body" and circulation apart from the areas of actual infection (Zone 1) and the areas of immune cell proliferation (Zone 2). The agents representing lymphocytes that have proliferated in Zone 2 and the granulocytes are the agent types found in Zone 3. The portal agents (Portals) in Zone 3 represent spatially discrete blood and lymphatic vessels controlling the access of the agents to Zone 1. They also transmit signals produced in Zones 1 and 2 to attract agents to migrate. The Portals also participate in the transportation of some signals to Zone 1. Portals are the means of transferring agents and signals from one zone to another. They are randomly placed in Zones 2 and 3. The variation and uncertainty in the time spent by immune cells in the areas represented by Zone 3 is one of the sources of randomness in the BIS.

The agents in BIS include: the dendritic cell agent (DC), the macrophage agent (MΦ), the natural killer agent (NK) of the innate immune system, the B cell agent (B), the T cell agent (T), and the cytotoxic lymphocyte T cell agent (CTL) of the adaptive immune system. The parenchymal cell agent (PC) in Zone 1 is replaced by the tumor cell agent (TumorAgent).

On the one hand, TumorAgents carry out proliferation, migration or quiescence according to the rules in **model of tumor growth**. On the other hand, they carry out the rules of PC in BIS. PCs infected by viruses have three possible outcomes:

① to be killed by NKs, pro-inflammatory T cell agents (T1s) or CTLs and undergo apoptosis;

② to be bound by antibodies (Ab) making them a target for recognition by pro-inflammatory macrophage agents (MΦ1s);

③to be lysed by complement products (C') if antibody 1 (Ab1) is present.

An attribute named *isSpectToLMP2 *is added to the CTL agent, which indicates whether the CTL is LMP2-specific. When a CTL makes contact with a TumorAgent, the attribute *effectiveContacts_CTL *of the latter will increase by *foldCTLSpectLMP2*; otherwise, it will increase by 1.

The basic idea of combining the tumor growth model and BIS in this paper is: TumorAgent grows in Zone 1. Concurrently, it is also infected with viruses that initiate defense by the immune system.

CTL immunotherapy is realized by infusing the CTL lines cultured in the laboratory into patients. In the simulation, this process is realized by adding CTLs into Zone 3. There are 100 Portals leading to Zone 1 in Zone 3. CTLs move randomly in Zone 3, and they enter Zone 1 when they meet a functional Portal.

In the first group of simulations, the treatment consists of two rounds of CTL addition into Zone 3. The number of CTLs added is fixed at 1800. The first round is at tick = 200, and the second is at tick = 200 ~ 254 with an interval of six ticks. This ensures that there are nine treatments with 10% of CTLs specific for LMP2. Ten simulations were carried out for each treatment.

In the second group of simulations, the time of CTL addition is fixed at tick = 200 and 242. The number of CTLs added is from 1000 to 2600 with an interval of 200. Ten simulations were carried out for each treatment.

The above simulation was repeated for 20% of CTL specific for LMP2.

The total numbers of tumor agents at tick = 200 and 350 were recorded for each simulation. The increment rate, k, obtained from the latter divided by the former, was also recorded; it reflects the therapeutic effect.

## Results

### The effect on EGFR of adding CTL agent

**Figure 1:**
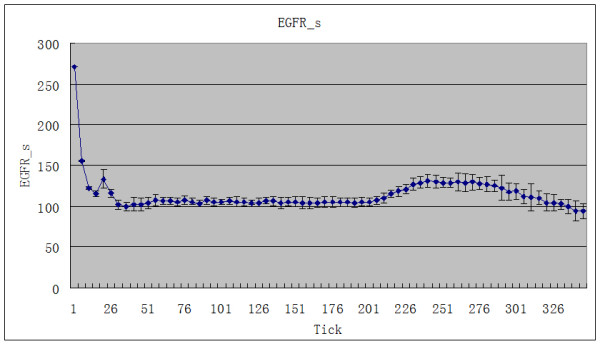
**The effect of adding CTL agent on EGFR_s**.

**Figure 2:**
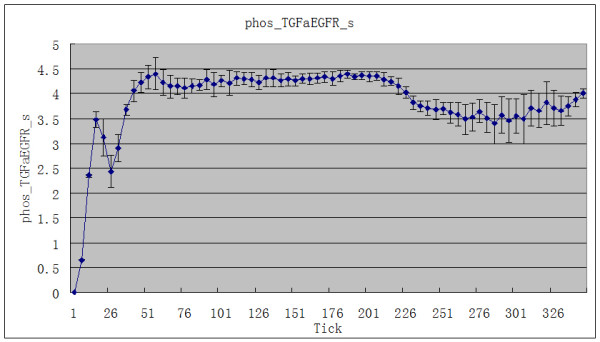
**The effect of adding CTL agent on phos_TGFaEGFR_s**.

The amount of CTL added was fixed at 1800. The two rounds of adding CTL were tick1 = 200 and tick2 = 242. Ten simulations were carried out. The average values and standard deviations of the EGFR concentration (EGFR_s) and the phosphorylated TGFα-EGFR complex concentration (phos_TGFaEGFR_s) in the tumor cell cytomembrane were calculated. The total number of tumor agents decreases after CTL is added. Corresponding to this, there is a rise in EGFR_s (Figure 1). Synchronously, there is a marked downward trend in phos_TGFaEGFR_s (Figure 2).

### Influence on therapeutic effect of the time-point of CTL addition and the percentage of LMP2-specific CTL

**Figure 3:**
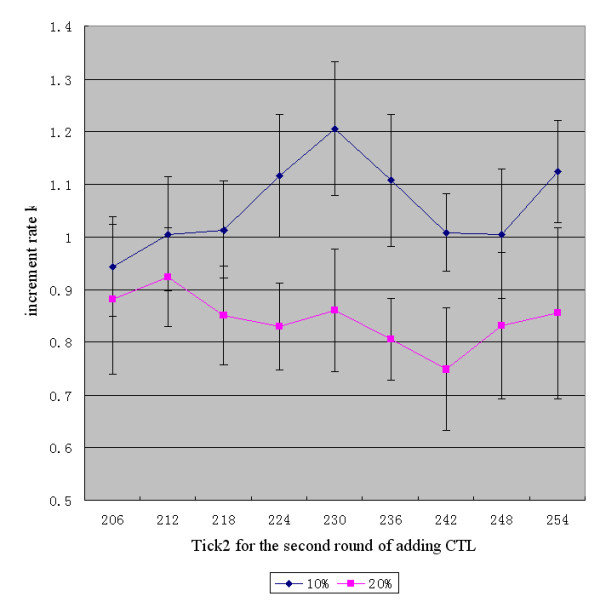
The specificity of CTL for LMP2: The relationship between the time points of CTL addition and the therapeutic effect $\left(\bar{x}\pm s\right)$

The amount of CTL added was fixed at 1800. The second round of CTL addition was from tick2 = 206 to 254 with an interval of six ticks. Ten simulations were carried out and the average value and standard deviation of increment rate, k, of tumor agents was calculated for each treatment. As shown in Table 1, the first row is for 10% of LMP2-specific CTL, and the second row is for 20% of LMP2-specific CTL. Figure 3 shows the data in Table 1 as a line chart.

**Table 1:** The relationships among the time-points of CTL addition, the percentage of LMP2-specific CTL and the increment rate k of tumor agents $\left(\bar{x}\pm s\right)$


**percentage of specific CTL**	**n**		**Tick2**								
		
			**206**	**212**	**218**	**224**	**230**	**236**	**242**	**248**	**254**

10%	10	$\bar{x}$	0.943	1.006	1.014	1.116	1.205	1.107	1.009	1.006	1.124
		
		***S***	0.094	0.108	0.092	0.116	0.176	0.125	0.073	0.123	0.098

20%	10	$\bar{x}$	0.881	0.924	0.851	0.830	0.861	0.806	0.749	0.832	0.856
		
		***S***	0.142	0.095	0.094	0.083	0.117	0.077	0.186	0.139	0.162

### Influence on the therapeutic effect of the amount of CTL added and the percentage of LMP2-specific CTL

**Figure 4:**
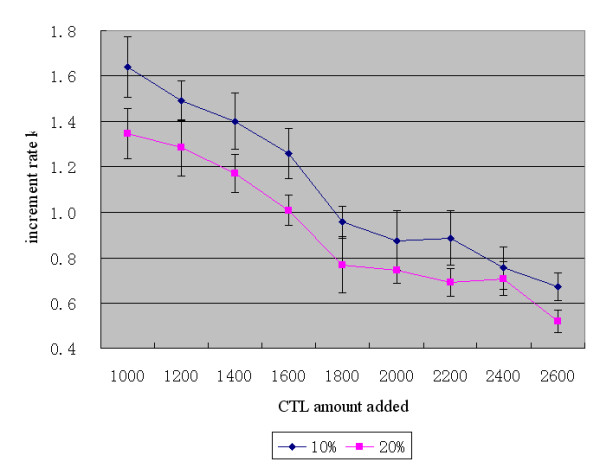
The specificity of CTL for LMP2: The relationship between amount of CTL added and the therapeutic effect $\left(\bar{x}\pm s\right)$

The time of adding CTL was fixed at tick = 200 and 242. The amount of CTL added was from 1000 to 2600 with an interval of 200. Ten simulations were carried out, and the average and standard deviation of the increment rate k of tumor agents were calculated for each treatment. As shown in Table 2, the first row is for 10% LMP2-specific CTL, and the second row is for 20% LMP2-specific CTL. Figure 4 shows the data in Table 2 as a line chart.

**Table 2:** The relationships among the amount of CTL added, the percentage of LMP2-specific CTL and the increment rate k of tumor agents $\left(\bar{x}\pm s\right)$


**percentage of specific CTL**	**n**		**CTL amount**								
		
			**1000**	**1200**	**1400**	**1600**	**1800**	**2000**	**2200**	**2400**	**2600**

10%	10	$\bar{x}$	1.640	1.493	1.402	1.260	0.956	0.876	0.887	0.755	0.673
		
		***S***	0.131	0.089	0.125	0.112	0.068	0.130	0.119	0.096	0.061

20%	10	$\bar{x}$	1.347	1.284	1.171	1.008	0.770	0.744	0.691	0.709	0.519
		
		***S***	0.111	0.123	0.085	0.066	0.123	0.055	0.062	0.074	0.049

### Validation of the model

**Figure 5:**
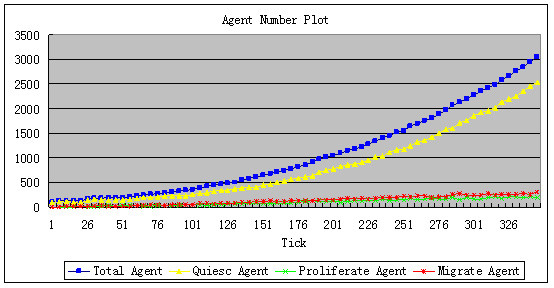
**The increase of tumor agent without CTL addition**. The blue curve gives the total number of tumor agents. The yellow, red and green curves are the numbers of agents in the quiescence, migration and proliferation states, respectively

**Figure 6:**
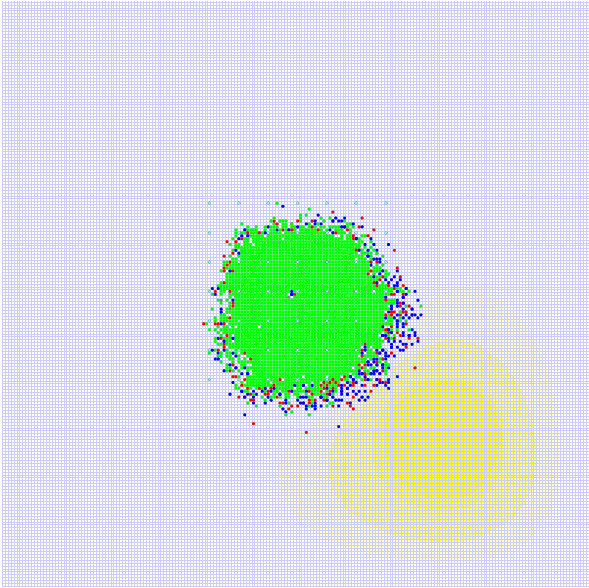
**The simulated result without adding CTL**. The green, blue and red dots represent tumor agents in the quiescence, migration, and proliferation states, respectively. The yellow shades are nutrient content, and the different shades of yellow denote different concentrations of nutrient

① Without addition of CTL, the total number of tumor agents shows exponential growth. As demonstrated in Figure 5, the blue curve gives the total number of tumor agents; the yellow, red and green curves are the numbers of agents in the quiescence, migration, and proliferation states, respectively. Figure 6 gives the overall scenario of tumor agents when the simulation is completed. The green, blue and red dots are agents in the quiescence, migration, and proliferation states, respectively. The yellow shades are nutrient content, and the different shades of yellow denote different concentrations of nutrient. The change in the shade of yellow indicates the diffusion of nutrient.

**Figure 7:**
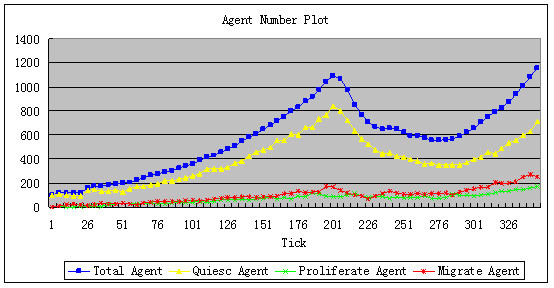
**The increase of tumor agent with CTL addition**. The blue curve gives the total number of tumor agents. The yellow, red and green curves are the numbers of agents in the quiescence, migration and proliferation states, respectively

**Figure 8:**
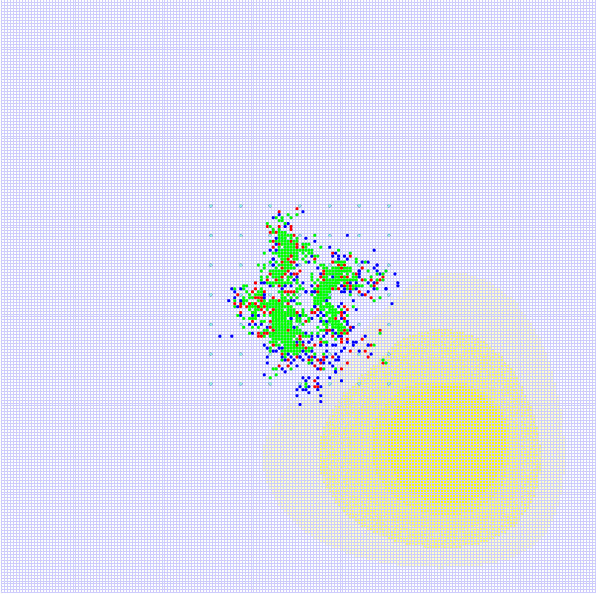
**The simulation result with CTL addition**. The green, blue and red dots represent tumor agents in the quiescence, migration, and proliferation states, respectively. The yellow shades are nutrient content, and the different shades of yellow denote different concentrations of nutrient

② To add CTLs, appropriate parameters need to be chosen. There is no significant change in the total number of tumor agents from time-point tick = 200 to tick = 350 (Figure 7), which corresponds to controlled tumor growth in clinical treatment. The simulation result with CTL added is shown in Figure 8. Major parameters used are listed in Table 3.

**Table 3:** Major parameters in the simulation


**Parameter**	**Annotation**	**value setting**

a1intAddCTLTick	The time at which the initial addition of CTL is made	200 ticks

a1intAddNumCTL	Number of CTL added for the first time	1800 ticks

a2intAddCTLTick	The time at which the second addition of CTL is made	206~254, the interval is 6 ticks

a2intAddNumCTL	Number of CTL added for the second time	1000~2600, the interval is 200 ticks

intSpectCTLPercent1	The percentage of CTL specific for LMP2	10% or 20%

## Discussion

The signal transduction network of the EGFR plays an important role in organ development, epithelization, regulation of cell growth, tumorigenesis and vascularization. Mutation, overexpression, structural rearrangement or loss of normal regulative function of EGFR lead to abnormal cell growth and differentiation [14]. Wang et al. [15] reported that the positive rate for EGFR was 70.9% in NPC tissue. EGFR expression was correlated with poorer overall survival (OS) and shorter time to progression (TTP). Yuan et al. [16] found that phosphorylated EGFR (pEGFR) was related to metastasis-free survival of NPC patients. The 5-year metastasis-free survival rate was significantly lower in patients with high pEGFR expression than in those with low pEGFR expression. Magkou et al. [17] found that pEGFR expression is related to angiogenesis and invasiveness and that the EGFR/pEGFR phenotype is associated with poor patient survival in invasive breast cancer. We found in the simulation that when CTL is added, the EGFR concentration in the tumor cell cytomembranes (EGFR_s) rises (Figure 1). Concurrently, there is a marked downward trend of active phosphorylated TGFα-EGFR complex (phos_TGFaEGFR_s, i.e. pEGFR) in those cytomembranes (Figure 2). This shows that tumor growth is suppressed and the activity of EGFR declines during CTL immunotherapy, which is consistent with the finding of Yuan et al. EGFR_s and phos_TGFaEGFR_s transform reciprocally, so the decline of the latter is followed by the rise of the former.

These simulations provide insights and clues for further clinical experimental research.

(1) The relationship between the CTL addition time-point and the therapeutic effect (Figure 3): According to the line chart for 10% LMP2-specific CTL, the increment rate k climbs from the minimum to the maximal value and then falls and reaches the lowest point at tick2 = 242 or 248. It begins to increase again at tick2 = 254. However, according to the line chart for 20% LMP2-specific CTL, k is lower than in the former case in general but the trend is similar, i.e., it reaches the upper point at tick2 = 230 and the lower point at tick2 = 242. This implies that a better therapeutic effect can be achieved here. According to the two graphs, better therapeutic effects can be achieved in both cases at tick2 = 242. One simulation time tick stands for the duration of one tumor cell proliferation event. This implies that a better therapeutic effect can be achieved when the CTL is added at 42 times the duration of one tumor cell proliferation step for both 10% or 20% LMP2-specific CTL, while other conditions are unaltered.

(2) The relationship between the amount of CTL added and the therapeutic effect (Figure 4): When the amount of CTL added is increased, the downward trend of k is similar for both 10% and 20% of LMP2-specific CTL. When the amount of CTL added increases from 1000 to 1800, k declines by 42.6% more quickly than usual. When the amount increases from 1800 to 2600, k declines by 30.9% relatively slowly. This suggests that increasing the amount of CTL added can improve the therapeutic effect, but the degree of improvement achieved with the same addition of CTL declines markedly after the amount added has increased to a certain level.

In this work, the simulation was conducted in silico. Although the pEGFR result is consistent with the finding of Yuan et al., the predicted relationship between treatment time-points, amount of CTL added, and the corresponding immunotherapeutic effect on NPC is rather difficult to validate in vivo. We expect interested researchers to carry out related laboratory validation studies. The agent-based quantitative simulation model built for CTL immunotherapy is an open model. The results from related laboratory studies in the future can gradually improve the model to attain more abundant, accurate and reliable quantitative simulation results to enhance the effect of immunotherapy for NPC in the clinic.

Fewer agents are used in this model than occur in vivo, both the tumor agents and the immune cells in BIS. The maximum number of tumor agents is 3000, which is still far less than in reality. Moreover, our simulation was carried out on a 2-dimensional model, and 3-dimensional simulation needs to be conducted in further studies. In two dimensions, the agents live in lattices located in one plane. This means that only one layer of agents is studied. Three-dimensional simulation is for multilayer agents and is more appropriate for modeling biological tissues.

## Competing interests

The authors declare that they have no competing interests.

## Authors' contributions

The work presented here was carried out in collaboration between all authors. DQK and LJM defined the research theme. WSJ, GYB and SLY designed the ABM model, carried out the simulations, analyzed the data, interpreted the results and wrote the paper. All authors read and approved the final manuscript.
